# Domain analysis of symbionts and hosts (DASH) in a genome-wide survey of pathogenic human viruses

**DOI:** 10.1186/1756-0500-6-209

**Published:** 2013-05-24

**Authors:** Mileidy W Gonzalez, John L Spouge

**Affiliations:** 1 National Institutes of Health, National Library of Medicine, National Center for Biotechnology Information, 8600 Rockville Pike, Building 38A, Room 6N611-M, Bethesda, MD 20894, USA

**Keywords:** Functional similarity, Domain similarity, Host-pathogen interactions, Gene transfer, Comparative genomics, Cellular homolog, Host-virus coevolution

## Abstract

**Background:**

In the coevolution of viruses and their hosts, viruses often capture host genes, gaining advantageous functions (*e.g.* immune system control). Identifying functional similarities shared by viruses and their hosts can help decipher mechanisms of pathogenesis and accelerate virus-targeted drug and vaccine development. Cellular homologs in viruses are usually documented using pairwise-sequence comparison methods. Yet, pairwise-sequence searches have limited sensitivity resulting in poor identification of divergent homologies.

**Results:**

Methods based on profiles from multiple sequences provide a more sensitive alternative to identify similarities in host-pathogen systems. The present work describes a profile-based bioinformatics pipeline that we call the Domain Analysis of Symbionts and Hosts (DASH). DASH provides a web platform for the functional analysis of viral and host genomes. This study uses Human Herpesvirus 8 (HHV-8) as a model to validate the methodology. Our results indicate that HHV-8 shares at least 29% of its genes with humans (fourteen immunomodulatory and ten metabolic genes). DASH also suggests functions for fifty-one additional HHV-8 structural and metabolic proteins. We also perform two other comparative genomics studies of human viruses: (1) a broad survey of eleven viruses of disparate sizes and transcription strategies; and (2) a closer examination of forty-one viruses of the order Mononegavirales. In the survey, DASH detects human homologs in 4/5 DNA viruses. None of the non-retro-transcribing RNA viruses in the survey showed evidence of homology to humans. The order Mononegavirales are also non-retro-transcribing RNA viruses, however, and DASH found homology in 39/41 of them. Mononegaviruses display larger fractions of human similarities (up to 75%) than any of the other RNA or DNA viruses (up to 55% and 29% respectively).

**Conclusions:**

We conclude that gene sharing probably occurs between humans and both DNA and RNA viruses, in viral genomes of differing sizes, regardless of transcription strategies. Our method (DASH) simultaneously analyzes the genomes of two interacting species thereby mining functional information to identify shared as well as exclusive domains to each organism. Our results validate our approach, showing that DASH has potential as a pipeline for making therapeutic discoveries in other host-symbiont systems. DASH results are available at http://tinyurl.com/spouge-dash.

## Background

Many species interact persistently in symbiosis through mutualistic, commensalistic, or parasitic relationships. Such symbiotic associations can lead to long histories of coevolution, promoting horizontal transfer of genes between the corresponding species. Acquired genetic material has afforded both prokaryotes and eukaryotes several advantageous new functions, including antibiotic resistance, nitrogen fixation, and even photosynthesis [1].

In the case of parasitic symbionts like viruses, most of the documented cases of gene transfer involve proteins with functions related to host immune system control or evasion. The large DNA viruses are particularly notorious for encoding homologs of cellular components of both the innate and adaptive arms of the immune response [2].

Homologs in host-virus systems have been traditionally identified [3-5] using pairwise sequence comparison methods like BLAST [6] and FASTA [7]. Yet, pairwise sequence comparison has limited sensitivity, particularly in detecting distant homologies. Profile sequence searches, which combine information from multiple sequences (*e.g*. PSSMs (Position Specific Scoring Matrices) [8,9], HMMs (Hidden Markov Models) [10-13]), have greater sensitivity than pair-wise sequence comparison in detecting distant homologs [9,14].

Profile search tools like PSI-BLAST and HMMER therefore provide more sensitive homology searches than BLAST or FASTA. Very few studies have investigated host-viral similarities (*e.g.*[15,16]) using profile search tools, however. Moreover, recent improvements to profile-based comparison algorithms have increased sensitivity further [17], thus improving their ability to identify even more distant homologies. In addition, previous studies of divergent host-viral similarities using profile search tools implemented their protocols as ad hoc solutions for specific viruses, thus, impeding automation and application in other viral systems.

The present article investigates functional similarity at the protein domain level by surveying similarities between the human genome and the genomes of an arbitrary but representative set of eleven viruses impacting human health. It also examines similarities between the human genome and the genomes of forty-one viruses of the order Mononegavirales. The functional comparisons are made with a bioinformatics pipeline that we call Domain Analysis of Symbionts and Hosts (DASH).

HHV-8 is the causative agent of Kaposi’s sarcoma, the most common AIDS-associated cancer [14], and it has also been associated with primary effusion lymphoma [15] and multicentric Castleman's disease [16]. Because many studies have documented a cellular origin for many genes in HHV-8 [2,13], HHV-8 provides an ideal model virus for validating our approach. This article therefore scrutinizes HHV-8 more closely than the other fifty-one viruses. Our comparative genomics survey includes DNA and RNA viruses of various genome sizes and transcription strategies, thereby providing a snapshot of the prevalence of functional similarities across a representative set of viruses impacting human health. The results for the fifty-two viruses surveyed here validate the methodology and show that DASH has potential as a pipeline for making therapeutic discoveries in other host-symbiont systems.

## Methods

### DASH’s computational pipeline

**Figure 1:**
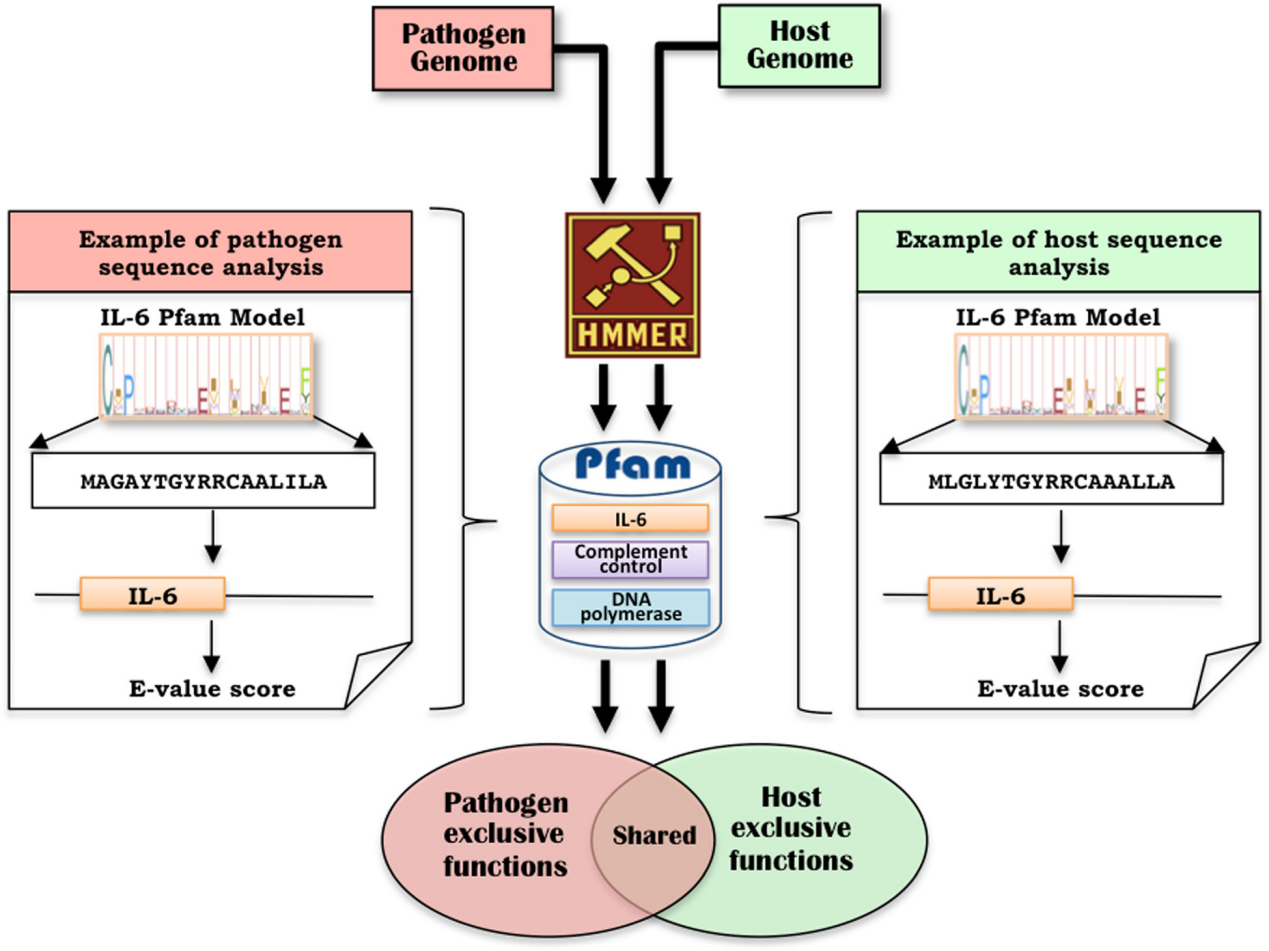
**DASH’s computational pipeline.** DASH compares a pathogen genome against the HMM profiles in Pfam using HMMER. As illustrated in the left-hand example, DASH compares all pathogen proteins against all PfamA profiles, annotating the domain function on the sequences and assigning E-value scores for the comparisons. All similarities with E-values under the user-defined threshold are reported as functional annotations in the pathogen genome. Likewise, DASH functionally annotates the host genome in parallel. With fully annotated genomes, DASH can distinguish the protein functions exclusive to the pathogen, functions exclusive to the host, and functions shared by both organisms.

**Figure 2:**
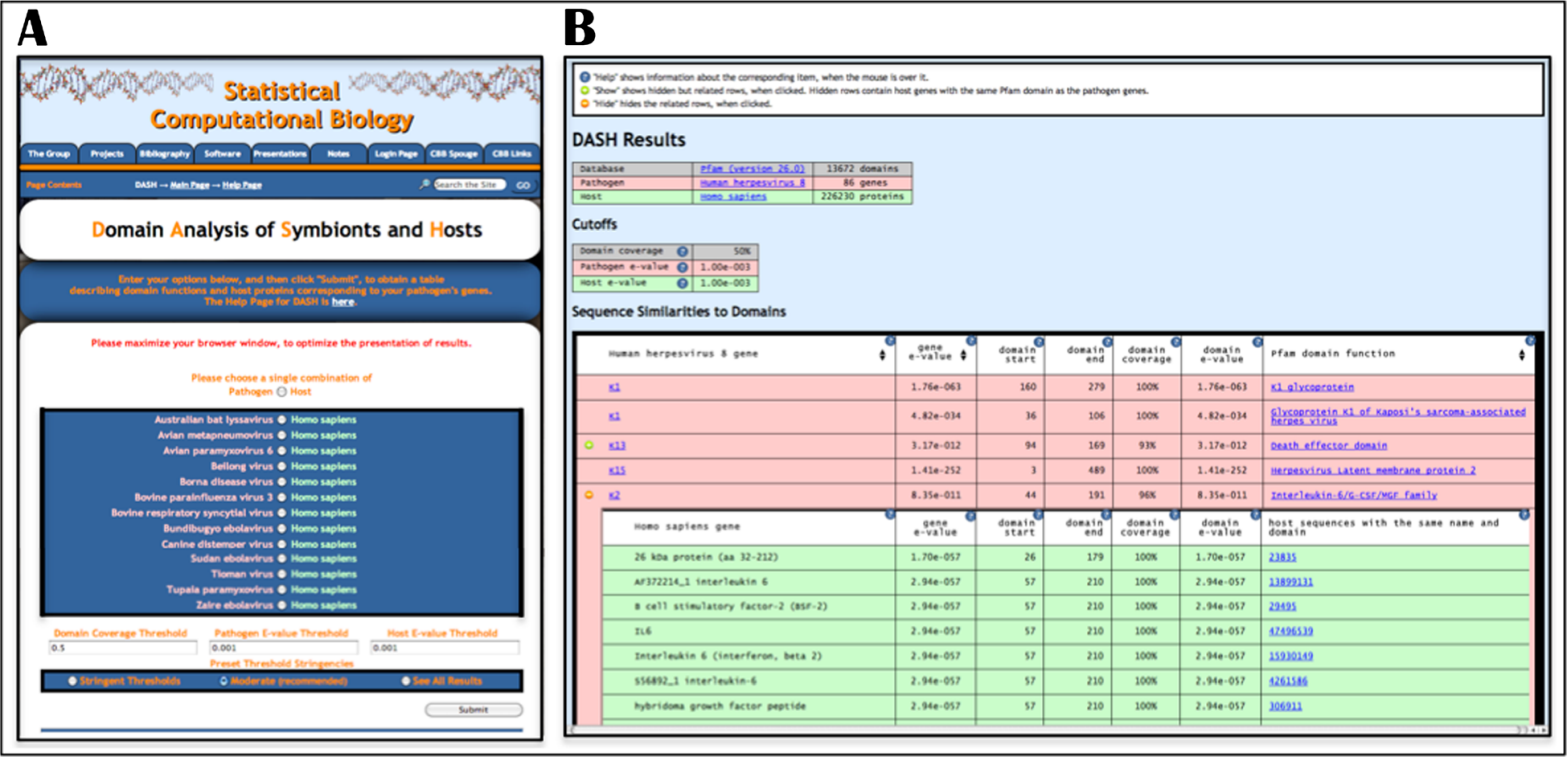
**DASH’s web interface. A**. DASH’s web interface currently allows the analysis of fifty-two host-virus systems, accepting user-defined scoring parameters or providing statistical guidance for choosing appropriate scoring thresholds. **B**. DASH results distinguish pathogen-exclusive from shared functions. The web interface permits rows corresponding to shared functions within the virus to be expanded to display the host sequences with the same function.

DASH compares each of the genes in a pathogen genome against a local collection of protein domain families (Figure 1). DASH performs the sequence comparisons using HMMER’s hmmscan v. 3.0 [17] against all the HMM profiles in the Pfam-A subset of Pfam v. 26 [18]. PfamA features 13,672 protein domain models, making it a relatively exhaustive repository, one manually built by experts from representative sets of sequences. DASH records all significant similarities to the Pfam models for each of the pathogen’s genes (Figure 1). A parallel analysis also functionally annotates the host genome (Figure 1). DASH distinguishes the protein domains exclusive to the host and pathogen from those shared between them (Figure 1, Figure 2).

### Detecting functional similarities between the human and viral genomes

DASH compared eighty-six proteins in the HHV-8 genome (reported by NCBI’s Refseq viral genome collection as of May 11, 2011) against the 13,672 PfamA v. 26 models. DASH also analyzed 226,230 human proteins from NCBI’s NR database (release date: February 2, 2012). The reported similarities for searches in both genomes were considered significant under a threshold E-value<1e-3 [17] after applying a Bonferroni multiple-test correction. The E-values in this study were multiple-test corrected to account for multiple comparisons (number of Pfam domain models times the number of proteins in the relevant organism, host or pathogen). When DASH matches a sequence to a Pfam domain model, it reports domain coverage, the fraction of the length of the domain model matched. To avoid spurious short sequence matches, this study reports a match only if the corresponding domain coverage exceeds 0.50. The same protocol described for the functional analysis of the Human and HHV-8 genomes was applied to analyze the eleven viruses in the comparative genomics survey and the forty-one viruses in the examination of the order Mononegavirales. The fifty-two viruses were extracted from the Refseq viral genome collection of May 11, 2011.

## Results and discussion

### DASH: An automated system for the whole-genome detection of functional similarity in host-symbiont systems

DASH automates the functional characterization of host and symbiont genomes through genome-wide profile-based similarity searches. By using HMMER’s hmmscan, DASH compares all pathogen proteins against all Pfam domain models thereby generating a list of putative functional annotations. Likewise, the pipeline functionally annotates the host proteome in parallel (Figure 1). All DASH output relevant to this paper is publicly available at http://tinyurl.com/spouge-dash.

DASH allows users to compare the functional annotations of two genomes simultaneously to distinguish shared protein domains from domains exclusive to each organism (Figure 2). The user can input specific scoring parameters or allow the system to provide statistical guidance for choosing appropriate scoring thresholds (Figure 2A). In its current prototype, DASH allows the analysis of fifty-two reference viral genomes. Plans for future releases include modifications to allow users to analyze custom sequences (*e.g.* different isolates of the same virus for intra-viral comparison).

The DASH output on the web classifies the functions into shared and pathogen-exclusive functions (Figure 2B). If DASH marked functional annotations on the pathogen as shared domains, the DASH output can be expanded to display information about their functional counterparts in the host (Figure 2B). In addition, the site has been linked to other in-house (*e.g.* NCBI taxonomy, NR) and external analysis resources (*e.g.* Pfam) to facilitate the exploration and functional characterization of pathogenic sequences.

### DASH confirms 24/25 human homologs in HHV-8

The HHV-8 genome contains eighty-six genes. Several studies have documented a cellular origin for 25/86 genes in HHV-8 [3,4,19-26]. DASH confirmed 24/25 previously reported homologies between HHV-8 and human (Table 1). Therefore, likely, the percent of the HHV-8 genome shared with its host is at least 29%.

**Table 1:** Functional similarities between HHV-8 and human confirmed by DASH

**HHV-8**				**Human**			**Function**	**PfamA accession**	**Function type**	**Original study**
**Gene**	**Protein gi**	**E-value**	**Dom. Cov.**	**Protein gi**	**E-value**	**Dom. Cov.**				
ORF9	139472809	2.12e-141	0.99	14250670	4.95e-134	0.97	DNA polymerase	PF00136, PF03104	M	Russo *et al.*
ORF61	139472880	6.11e-124	1.00	194389080	7.11e-211	1.00	Ribonucleotide reductase, large chain	PF02867, PF00317	M	Russo *et al.*
ORF70	139472812	3.29e-109	1.00	4507751	2.69e-113	1.00	Thymidylate synthase	PF00303	M	Russo *et al.*
ORF60	139472853	2.23e-87	0.97	4557845	1.79e-116	1.00	Ribonucleotide reductase, small chain	PF00268	M	Russo *et al.*
ORF75	139472887	1.76369e-42	0.99	38197270	9.58835e-88	1.00	CobB/CobQ-like glutamine amidotransferase, AIR synthase	PF13507, PF02769, PF12818	M	Russo *et al.*
ORF2	139472863	5.06e-22	0.98	4503323	6.19e-25	0.99	Dihydrofolate reductase	PF00186	M	Russo *et al.*
ORF72	139472885	3.41e-21	0.98	4502613	3.71e-39	0.99	Cyclin	PF00134, PF09080	I	Russo *et al.*
K9	139472878	2.00e-16	0.74	119604110	2.26e-59	0.99	Interferon regulatory factor	PF10401, PF00605	I	Russo *et al.*
ORF46	139472838	6.58e-15	0.97	194387970	8.97e-20	0.97	Uracil DNA glycosylase	PF03167	M	Russo *et al.*
ORF74	139472805	4.12e-14	1.00	197692661	5.57e-94	1.00	7 transmembrane receptor	PF00001	M	Russo *et al.*
K4	139472814	1.18e-13	0.95	2905626	3.40e-15	0.95	Small cytokine, interleukin-8 like	PF00048	I	Russo *et al.*
K6	139472866	1.88e-13	0.94	2905626	3.40e-15	0.95	Small cytokine, interleukin-8 like	PF00048	I	Russo *et al.*
ORF56	139472847	1.07e-12	0.97	119625077	1.42e-07	0.99	Herpesviridae UL52/UL70 DNA primase	PF03121	M	Russo *et al.*
ORF71/K13	139472802	3.17e-12	0.93	194386924	1.55e-18	0.99	CASP8 and FADD-like apoptosis regulator	PF01335	I	Russo *et al.*
K2	139472811	8.35e-11	0.96	23835	1.70e-57	1.00	Interleukin-6/G-CSF/MGF family	PF00489	I	Russo *et al.*
ORF54	139472845	9.88e-08	0.78	158256462	7.73e-40	0.99	dUTPase	PF00692	M	Russo *et al.*
K5	139472815	6.46686e-07	1.00	221043596	1.14442e-06	1.00	Membrane-Associated RING-CH (MARCH)	PF12906	I	Bartee *et al.*
K3	139472864	2.58674e-06	1.00	221043596	1.14442e-06	1.00	Membrane-Associated RING-CH (MARCH)	PF12906	I	Bartee *et al.*
K4.1	139472813	3.06e-06	0.91	2905626	3.40e-15	0.95	Small cytokine, interleukin-8 like	PF00048	I	Neipel *et al.*
ORF16	139472817	3.64e-05	0.95	10279702	3.71e-24	1.00	Apoptosis regulator, Bcl-2 family	PF00452	I	Russo *et al.*
vIRF-3/K10.5/K10.6	139472850	1.13e-03	0.68	119604110	2.26e-59	0.99	Interferon regulatory factor	PF10401	I	Russo *et al.*
KCP/ORF4	139472860	1.29e-02	0.91	80478231	1.42e-04	1.00	Complement control protein	PF00084	I	Russo *et al.*
vIRF-2/K11/K11.1	139472851	6.35e-01	0.68	119604110	2.26e-59	0.99	Interferon regulatory factor	PF10401	I	Russo *et al.*
vIRF-4/K10/K10.1	139472849	4.94e+00	0.76	119604110	2.26e-59	0.99	Interferon regulatory factor	PF10401	I	Russo *et al.*

Abbreviation: (Dom Cov.) Domain coverage—fraction of Pfam HMM model coverage by viral/human sequence alignment. Function types: (I) Immunomodulatory, and (M) metabolic.

Of the twenty-four HHV-8 genes DASH identifies as cellular homologs, fourteen genes feature immunomodulatory and ten metabolic functions. DASH misses the putative homology between ORF63 and human NLRP that Gregory *et al.* reported as having a blastp E-value=2e-4 [27]. Although we attempted to reproduce the results of Gregory *et al****.*** with DASH and blastp, we were unable to find significant similarities between ORF63 and NLRP. It appears that the marginal E-value observed by Gregory *et al.* was obtained using blast2seq, which compares a single query against only a single subject sequence. Blast2seq calculates E-values using a database length equal to the length of only one sequence (opposed to using the length of thousands of sequences). Thus, the difference in our E-values is likely the result of differing database sizes, and therefore dependent on the relevant multiple-test correction. Gregory *et al.* do adduce experimental evidence to validate the functional similarities between the two proteins, however.

When coupling computational functional analyses on HHV-8 with manual searches of the literature, it was reassuring to learn that our results agreed with previous experimental reports. Computational methods alone were able to repeat previous findings of functional similarity identified between HHV-8 and human, thereby validating our approach.

DASH also detected that 51/86 genes in HHV-8 have functions exclusive to the virus (Table 2). DASH confirmed previously published annotations for 30/51 HHV-8 genes as viral structural/metabolic proteins. The remaining 21/51 genes in Table 2 are viral-exclusive genes displaying conserved domains with unknown functions.

**Table 2:** Exclusively-viral functions identified by DASH in the HHV-8 genome

**Gene**	**Protein gi**	**E-value**	**Dom. Cov.**	**Function**	**PfamA accession**	**Function type**	**Reference**
ORF25	139472823	0.0	1.00	Herpes virus major capsid protein	PF03122	S/M	Russo *et al.*
ORF44	139472836	0.0	1.00	Helicase	PF02689	S/M	Russo *et al.*
ORF6	139472807	0.0	0.98	ssDNA binding protein	PF00747	S/M	Russo *et al.*
ORF8	139472808	5.6e-272	0.99	Glycoprotein B	PF00606	S/M	Russo *et al.*
ORF63	139472855	1.4e-271	1.00	Herpes virus tegument protein U30	PF04523	S/M	Russo *et al.*
K15	139472806	1.2e-258	1.00	Herpesvirus Latent membrane protein 2	PF06126	S/M	Choi *et al.*
ORF24	139472822	2.6e-208	1.00	Herpesvirus UL87 family	PF03043	C	DASH
ORF43	139472835	1.1e-200	1.00	Herpesvirus UL6 like	PF01763	C	DASH
ORF22	139472821	9.1e-197	1.00	Herpesvirus glycoprotein H	PF02489	S/M	Russo *et al.*
ORF50	139472840	1.9e-193	1.00	Transcription activation factor (transactivator)	PF03326	S/M	Russo *et al.*
ORF7	139472861	7.5e-193	1.00	Herpesvirus processing and transport protein	PF01366	S/M	Russo *et al.*
ORF19	139472819	7.2e-182	1.00	Herpesvirus UL25 family	PF01499	C	DASH
K8	139472841	1.3e-170	1.00	Transcriptional activator	PF07188	S/M	Wu *et al.*
ORF37	139472831	3.0e-164	0.99	Alkaline exonuclease	PF01771	S/M	Russo *et al.*
ORF29	139472824	1.1e-154	1.00	Probable DNA packing protein	PF02499, PF02500	S/M	Russo *et al.*
ORF59	139472852	2.2e-139	1.00	Herpes DNA replication accessory factor	PF04929	S/M	Russo *et al.*
ORF39	139472832	6.3e-136	0.96	Herpesvirus glycoprotein M	PF01528	S/M	Russo *et al.*
ORF68	139472883	7.6e-133	0.99	Herpesvirus putative major envelope glycoprotein	PF01673	S/M	Russo *et al.*
ORF58	139472879	5.2e-128	1.00	Herpesvirus BMRF2 protein	PF04633	S/M	Russo *et al.*
ORF49	139472877	3.3e-108	1.00	BRRF1-like protein	PF04793	S/M	Russo *et al.*
ORF34	139472829	1.6e-106	0.99	UL95 family	PF03038	C	DASH
ORF26	139472871	3.1e-103	1.00	Herpesvirus VP23 like capsid protein	PF01802	S/M	Russo *et al.*
ORF18	139472818	8.6e-99	1.00	UL79 family	PF03049	C	DASH
ORF23	139472870	1.1e-98	1.00	Herpesvirus BTRF1 protein conserved region	PF04682	C	DASH
ORF10	139472862	6.3e-94	0.99	Herpesvirus dUTPase protein	PF04797	S/M	McGeehan *et al.*, Davidson *et al.*
ORF11	139472810	5.2e-93	0.99	Herpesvirus dUTPase protein	PF04797	S/M	McGeehan *et al.*, Davidson *et al.*
ORF69	139472884	3.5e-91	0.97	Herpesvirus UL31-like protein	PF02718	C	DASH
ORF55	139472846	3.9e-90	0.96	Herpes virus U44 protein	PF04533	C	DASH
ORF32	139472827	4.2e-84	1.00	Herpesvirus UL17 protein	PF04559	C	DASH
ORF66	139472857	3.9e-82	0.99	UL49 family	PF03117	C	DASH
ORF62	139472854	4.7e-81	1.00	Herpesvirus capsid shell protein VP19C	PF03327	S/M	Russo *et al.*
ORF48	139472839	4.4e-78	1.00	Herpesvirus protein of unknown function	PF05734	C	DASH
ORF17	139472867	2.2e-77	0.65	Assemblin (Peptidase family S21)	PF00716	S/M	Unal *et al.*
ORF31	139472825	8.0e-71	0.98	UL92 family	PF03048	C	DASH
K1	139472859	1.5e-69	1.00	Glycoprotein	PF02960, PF11049	S/M	Lee *et al.*
ORF67	139472856	1.7e-69	0.97	Herpesvirus virion protein U34	PF04541	C	DASH
ORF33	139472828	2.4e-69	0.99	Herpesvirus UL16/UL94 family	PF03044	C	DASH
ORF64	139472881	1.3e-61	1.00	Herpesvirus tegument protein	PF04843	S/M	Russo *et al.*
ORF20	139472820	2.1e-61	0.99	Herpes virus protein UL24	PF01646	C	DASH
ORF65	139472858	2.2e-59	1.00	Gammaherpesvirus capsid protein	PF06112	S/M	Russo *et al.*
ORF42	139472834	5.3e-56	1.00	Herpesvirus UL7 like	PF01677	C	DASH
ORF57	139472848	1.4e-54	0.99	Herpesvirus transcriptional regulator	PF05459	S/M	Kirshner *et al.*
ORF35	139472830	1.6e-47	0.99	Gammaherpesvirus protein of unknown function	PF05852	C	DASH
ORF40	139472833	4.2e-46	1.00	DNA helicase/primase	PF05774, PF03324	S/M	Russo *et al.*
ORF47	139472876	6.3e-36	0.95	Viral glycoprotein L	PF11108	S/M	Russo *et al.*
ORF52	139472843	5.1e-35	1.00	Herpesvirus BLRF2 protein	PF05812	S/M	Russo *et al.*
ORF53	139472844	1.7e-28	0.99	UL73 viral envelope glycoprotein	PF03554	C	DASH

Abbreviation: (Dom Cov.) Domain coverage—fraction of Pfam HMM model coverage by viral/human sequence alignment. Function types: (S/M) Structural/Metabolic, (C) Conserved unknown.

Because the similarities listed in Table 1 and Table 2 have highly significant E-values, they are likely true homologs. Protein domain sequence similarities at E-value <1e-03 are widely-accepted as being significant [17]; indeed, scores approximating this threshold are expected in cases of distant similarities. Thus, the homologies reported here become more compelling still, given that even after the multiple-test correction, for most of the proteins the E-values are much smaller than the accepted 1e-03 threshold. Table 1 lists 21/24 shared protein domains with multiple-test-corrected E-values ranging from 2.1e-141 to 1.1e-3 in HHV-8 (and 7.1e-211 to 1.4e-04 in human). Because of multiple-test correction, 3/24 HHV-8 homologs fall below accepted thresholds of similarity however. Table 2 shows fifty-one viral-exclusive genes with E-values ranging from 0.0 to 5.7e-15. As additional evidence of the reported functional similarities, ninety-five of the homologs shown in Table 1 and Table 2 have a domain coverage >0.75 (lowest overall domain coverage = 65). The high fraction of domain coverage suggests that the domains are largely complete and are thus functional instances (*i.e.* working opposed to degraded copies) of the domain.

### DASH identifies homologies between humans and both the DNA and RNA viruses

To measure the prevalence of protein domain similarities between human and various types of viruses, we used DASH to survey the eleven viruses in Table 3, which have implications for human health. To make the survey as broad as possible, the viruses in Table 3 were selected to have disparate genome sizes and to represent varying classes of viruses (five DNA viruses and six RNA viruses; four retro-transcribing viruses and seven non-retro-transcribing viruses).

**Table 3:** A genome-wide comparative survey of human viruses of disparate sizes and transcription strategies

**Virus name**	**Virus type**	**Viral transcription**	**Protein-coding genes**	**Shared genes**	**% Genome shared**
Human herpesvirus 8	DNA	Non-retro-transcribing	86	25	29.1
Human parvovirus B19	DNA	Non-retro-transcribing	3	0	0.0
Human herpesvirus 4	DNA	Non-retro-transcribing	94	12	12.8
Human herpesvirus 5	DNA	Non-retro-transcribing	165	14	8.5
Hepatitis B virus	DNA	Retro-transcribing	7	1	14.3
Human T-lymphotropic virus 1	RNA	Retro-transcribing	11	6	54.5
Human immunodeficiency virus 1	RNA	Retro-transcribing	27	11	40.7
Human T-lymphotropic virus 2	RNA	Retro-transcribing	10	3	30.0
Zaire ebolavirus	RNA	Non-retro-transcribing	9	0	0.0
Rotavirus A	RNA	Non-retro-transcribing	12	0	0.0
Influenza A virus (A/Puerto Rico/8/34(H1N1))	RNA	Non-retro-transcribing	13	0	0.0

The present study confirms previous reports of cellular homologies in the DNA viruses (detailed lists of the similarity hits in Table 3 can be found at http://tinyurl.com/spouge-dash). DASH detects human homologs in all but one of the DNA viruses tested (4/5), both large and small. In the DNA viruses, the percent of viral genome shared with the host ranges from 0% in Human parvovirus B19 to 29% in HHV-8. Thus, HHV-8 appears to share a relatively large percentage of its genome with its host, compared to the rest of the DNA viruses examined.

The retro-transcribing RNA viruses in Table 3 displayed larger fractions of functional similarities to humans than any of the DNA viruses. The retroviruses analyzed share 30% (HTLV-2) to 55% (HTLV-1) of their genomes with their human host. The non-retro-transcribing RNA viruses in Table 3 showed no evidence of homology to humans, however.

Sequence similarity methods such as the one used by DASH cannot alone assign a definitive directionality to gene transfer between a pair of organisms with similar proteins. Nonetheless, DASH provides useful evidence suggesting similar directionality tendencies for the human DNA and RNA retro-transcribing viruses as those identified previously with other methods.

Most of the domain similarities DASH identifies in the DNA viruses feature domains involved in cellular (opposed to viral) processes (*e.g.* apoptosis regulation, cytokine signaling), in accord with the notion that cellular homologs in the DNA viruses are the result of molecular mimicry by the viruses to subvert the host immune system [3,28-30]. In contrast, the annotations on the homologies between RNA retro-transcribing viruses and humans suggest retroviruses as donors of the shared genetic material, because most of the domains common to both allude to viral functions (*e.g.* integrase, retroviral aspartyl protease). The predominance of retrovirus-to-host transfer is consistent with the knowledge that endogenous retroviral genes constitute 7-8% of the human genome [31].

### Homologies between humans and non-retro-transcribing RNA viruses

Non-retro-transcribing RNA viruses have no obvious means of capturing DNA from their host. To examine horizontal gene transfer in a non-retro-transcribing RNA viral order and the corresponding hosts, we used DASH to analyze all the human mononegaviruses in the NCBI repository.

**Figure 3:**
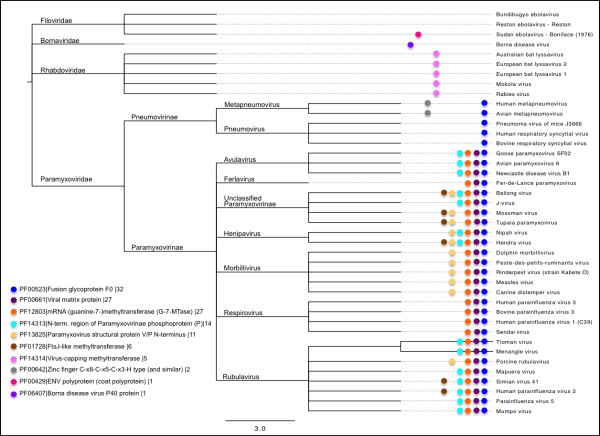
**Functional domains shared by humans and forty-one RNA non-retro-transcribing viruses.** A species tree of the order Mononegavirales from the NCBI taxonomy. Colored dots on the tree show the mononegaviruses with homologous domains in human. The lower left-hand side of the diagram maps colors to functional domains in the form of [color]|[PfamA accession]|[functional domain description]|[number of mononegaviruses showing given function].

DASH detects homologs between 39/41 mononegaviruses and human (Figure 3 and Table 4). The percent of viral genome shared with the host in the thirty-nine mononegaviruses ranged from 8% (Pneumonia virus of mice) to 75% (Hendra virus), with a median of 44%. Based on the results of Table 4, the median non-retro-transcribing RNA virus shows higher fractions of homologous host genes than the median DNA virus (11%) or median retrovirus (41%) from Table 3.

**Table 4:** Prevalence of homology to human genes in the non-retro-transcribing RNA viral order mononegavirales

**Virus name**	**Protein-coding genes**	**Shared genes **^**a**^	**% Genome shared **^**a**^
Hendra virus	8	6	75.0
Human parainfluenza virus 2	7	5	71.4
Simian virus 41	7	5	71.4
Goose paramyxovirus SF02	6	4	66.7
Newcastle disease virus B1	6	4	66.7
Mossman virus	8	5	62.5
Nipah virus	8	5	62.5
Tupaia paramyxovirus	8	5	62.5
Avian paramyxovirus 6	7	4	57.1
Canine distemper virus	7	4	57.1
Dolphin morbillivirus	7	4	57.1
Menangle virus	7	4	57.1
Rinderpest virus (strain Kabete O)	7	4	57.1
Beilong virus	11	6	54.5
Bovine parainfluenza virus 3	6	3	50.0
Measles virus	8	4	50.0
Mumps virus	8	4	50.0
Parainfluenza virus 5	8	4	50.0
Peste-des-petits-ruminants virus	8	4	50.0
Tioman virus	8	4	50.0
Mapuera virus	9	4	44.4
Porcine rubulavirus	9	4	44.4
Fer-de-Lance paramyxovirus	8	3	37.5
Human parainfluenza virus 3	8	3	37.5
J-virus	11	4	36.4
Human parainfluenza virus 1	10	3	30.0
Sendai virus	10	3	30.0
Avian metapneumovirus	9	2	22.2
Human metapneumovirus	9	2	22.2
Australian bat lyssavirus	5	1	20.0
European bat lyssavirus 1	5	1	20.0
European bat lyssavirus 2	5	1	20.0
Mokola virus	5	1	20.0
Rabies virus	5	1	20.0
Borna disease virus	6	1	16.7
Sudan ebolavirus	8	1	12.5
Bovine respiratory syncytial virus	11	1	9.1
Human respiratory syncytial virus	11	1	9.1
Pneumonia virus of mice J3666	12	1	8.3
Bundibugyo ebolavirus	9	0	0.0
Reston ebolavirus	8	0	0.0

^a^ Refer to http://tinyurl.com/spouge-dash for full genome annotations for each virus including lists of viral-exclusive and shared genes.

Figure 3 maps the homologies DASH identifies between humans and thirty-nine mononegaviruses onto the mononegaviral species tree. Several of the homologous domains in Figure 3 are present in complete taxa. For instance, PF12803 is present in all the Paramyxovirinae, while PF14314 is conserved in all the Rhabdoviridae. DASH also confirmed the human homologs in Bornaviridae and Filoviridae identified by [32,33] as showing non-retrotranscribing RNA viruses as contributors to the make-up of vertebrate genomes (Figure 3 and Table 5).

**Table 5:** Summary of homologous domains between humans and forty-one mononegaviruses

**Pfam domain**	**Domain name**	**Function**	**Viral**		**Human**			**Human homologs**
			**E-value range**	**Median domain coverage**	**E-value range**	**Median domain coverage**	**Human seq. hits per virus**	
PF06407	Borna disease virus P40	Target for MHC class I-restricted cytotoxic T-cell response	2.4e-266 - 2.4e-266	1.00	4.6e-7 - 2.5e-77	0.52	6	Endogenous, Bornavirus-like nucleoprotein 1, Endogenous Bornavirus-like nucleoprotein 2
PF01728	FtsJ-like methyltransferase	Methyltransferase	7.8e-4 - 1.8e-6	0.52	6.6e-6 - 2.4e-66	0.65	43	Many ^c^
PF14314	Virus-capping methyltransferase	Virus-capping methyltransferase	4.1e-84 - 2.9e-106	0.98	5.1e-04 - 5.1e-04	0.10	1	Hypothetical protein
PF00642	Zinc finger	Mediates binding specificity	4.5e-05 - 4.5e-05	0.89	1.0e-3 - 6.5e-13	0.93	294	Many ^c^
PF12803 ^a^	mRNA methyltransferase	Cap methylation of mRNA.	9.0e-93 - 4.6e-117	1.00	1.4e-4 - 2.5e-4	0.37	3	Putative ADP-ribosylation factor-like protein 5C, hCG31412, isoform CRA_a, hCG31412, isoform CRA_b
PF00429 ^a^	ENV polyprotein (coat polyprotein)	Coat polyprotein	1.0e-03 - 1.0e-03	0.17	9e-4 - 1.6e-68	0.27	51	Many ^c^
PF00523^b^	Fusion glycoprotein (F0)	Viral attachement	7.4e-96 – 8.8e-189	1.00	1.4e-64 - 1.4e-64	0.44	1	Angrgm-52
PF00661 ^b^	Viral matrix protein (M)	Viral assembly	4.1e-84 – 2.3e-146	0.99	2.9e-106 - 2.9e-106	0.37	1	Angrem52
PF14313 ^b^	N-terminal Paramyxovirinae phosphoprotein (P)	Phosphoprotein	4e-4 - 2e-26	0.95	1.4e-21 - 1.4e-21	0.98	1	AngRem104
PF13825 ^b^	N-terminal Paramyxovirus structural protein (V/P)	Structural protein	7.6e-4 - 5.4e-153	0.75	2.2e-4 - 2.2e-4	0.42	1	AngRem104

^a^ Similarities with weaker evidence (*i.e.* high E-values, low domain coverage, numerous human sequence hits).

^b^ Similarities likely resulting from mis-annotation of human sequences.

^c^ Refer to http://tinyurl.com/spouge-dash for detailed lists of the similarity hits for individual viruses.

Inferring homology from significant sequence similarity has been a routine bioinformatics practice since the 1990’s. Homology can be reliably inferred for proteins sharing statistically significant sequence similarity [34], permitting inferences about the structure and function of unknown molecules with characterized homologs. Sequencing and annotation errors, however, can mislead homology inference.

We identified an example of DASH's susceptibility to annotation errors in the putative homologies it reported for some mononegaviruses. DASH identified two human proteins (AngRem104 and AngRem52) as significantly similar to the F, M, V, and P genes of the paramyxoviruses in the order Mononegavirales (Table 5). A 2003 study annotated AngRem104 and AngRem52 in the public databases as the products of two human genes upregulated by Angiotensin II in mesangial cells [35]. Yet, later studies demonstrated that AngRem104 and AngRem52 were actually proteins coded by two new paramyxoviruses [36,37]. Thus, it appears that AngRem104 and AngRem52 are not human, but viral homologs of paramyxoviruses. Table 5 flags domains unreliable because of possible sequence misannotations.

We also caution DASH users to consider E-values, domain coverage, and the number of similarity hits on the host and virus when evaluating putative homologies. For instance, the human homologs of mRNA methyltransferase (PF12803) in Table 5 have two weak similarity descriptors, namely marginal E-values and low-domain coverage. Weak similarity descriptors do not invalidate homology candidates. Low domain coverage in a true homolog could indicate a partial domain, while marginal E-values could indicate a distant homology. Instead, weak descriptors caution further investigation. Table 5, e.g., flags the similarities to (PF00429) as providing only weak evidence.

### DASH reports a subset of non-retrotranscribing viruses with no homology to humans

The apparent lack of homology between human genes and those of some non-retro-transcribing viruses (Table 3 and Table 4) has several possible causes. Perhaps, a molecular mechanism peculiar to their unique biology has prevented gene transfer between humans (or their ancestors) and non-retro-transcribing viruses. Although no clear evidence of recombination has been detected for non-retro-transcribing viruses [38], vertebrate genomes have endogenized some non-retro-transcribing viral elements [32,33]. Table 4 and Table 5 therefore suggest that humans (or their ancestors) might have endogenized elements from the majority of human mononegaviruses.

Although most (if not all) non-retro-transcribing viruses lack a mechanism to integrate host genes, our methods did detect homologies between humans and mononegaviruses. Our methods cannot speak directly to the mechanism by which a homology is present or absent, although undetected homologies might always be a consequence of excessive sequence divergence. The genes of the non-retro-transcribing viruses for which no human similarity was evident may simply have evolved too far for our methods to detect the corresponding homology. Significant sequence similarity indicates homology; but lack of sequence similarity does not rule it out.

### Our approach to the detection of functional similarities in host-pathogen interactions

DASH provides a framework to automate the sequence analysis of the complete genomes of two interacting species (*e.g.* a host and a pathogen), because it does not depend on curating or creating multiple alignments of orthologous genes to identify homology. Instead, the multiple alignments are implicit in the functional domains modeled by Pfam.

DASH identifies similarities between the pathogen and host sequences at the protein domain level. By targeting domain-based similarities, the sequence searches gain sensitivity, particularly in cases where only part of the protein is conserved. Consider, e.g., the cytokine receptors acquired by poxviruses. The cellular homologs of the tumor necrosis factor receptor and of the gamma-interferon receptor in myxoma virus, as well as the IL-1 receptor homolog in vaccinia lack the membrane anchor and the cytoplasmic signaling domains. By coding only the ligand-binding domain, the viral homologs remain soluble, which, in turn, increases virulence since each can bind and neutralize the corresponding host cytokine, preventing the cellular receptors from delivering an antiviral signal [29]. Domain-restricted homology in viruses is therefore commonplace, particularly when the cellular homolog is a receptor or membrane-bound protein [29]. Domain-based sequence searches are particularly likely to detect these domain-restricted homologies, especially in cases of divergent similarities.

Previous experience informed our decision to use HMMER, instead of other profile-based sequence comparison methods such as PSI-BLAST. HMMER has been shown to be less susceptible to profile corruption, tends to have a higher sensitivity, and its programs are more amenable to searching against Pfam models [39]. In addition, by searching against curated functional models instead of building them iteratively DASH takes advantage of the transitivity of homology to identify more divergent similarities. DASH does not attempt to establish homology between the symbiont and the host sequence directly. Rather, by transitivity, it identifies whether two genes, one from a symbiont and one from a host, share homology to a common HMM profile before it reports them as functional similarity candidates.

In contrast, methods like PSI-BLAST first search a comprehensive protein sequence library. PSI-BLAST then builds a profile progressively over several search iterations, attempting to create an alignment phylogenetically diverse enough to detect host proteins homologous to a viral query protein, or vice versa. By taking advantage of the transitivity of homology, DASH exploits pre-computed, curated sequence alignments, identifying distant functional similarities more systematically. See Additional file 1: Table S1 for a comparison of the results obtained with PSI-BLAST when searching with the viral genes in Table 1.

Thus, the present study might have been successful, because it avoids *ad hoc* iterative homology searches. In iterative homology searches, if a viral sequence is too distant from the other members of its functional family, an iterative profile might lack sensitivity because it recruits too few close homologs of the viral sequence or does not weight them heavily enough. In contrast, experts have manually built Pfam profiles from representative sets of sequences, so its profiles are usually weighted evenly across the phylogenetic tree of the functional family. Moreover, Pfam features ~14,000 different functions, making it a relatively exhaustive repository of functional domains.

### Limitations of our approach

DASH requires sequenced genomes for both organisms in the analysis. Although DASH can analyze partial sequences, the resulting coverage will depend on the quantity and quality of sequences available. Fortunately, sequence data is widely available and, often, it is the only information available for newly identified organisms.

Likewise, the availability and phylogenetic breadth of the domain models in Pfam can limit DASH’s approach: if the HMM profiles searched do not include a given function, the method cannot indicate the corresponding functional similarity. Similarly, the method will experience limitations anytime the given HMM does not represent the organisms adequately (*e.g*. when the model is not phylogenetically broad enough to include them). But, we expect these limitations to be neither frequent nor severe, especially since DASH uses Pfam, which is an extensive database. Missing functions should be the exception and not the norm.

As for any method relying on sequence data, DASH is susceptible without much warning to sequencing and annotation errors. Table 4 and Table 5, e.g., show an annotation error in the analysis of the mononegaviruses. Public sequence databases seldom correct such annotation errors. Therefore, investigators should consider the similarities identified by DASH as helpful but still controvertible evidence of putative homologies, meriting further investigation as biological interest indicates.

## Conclusions

The present article described a genome-wide survey of protein domain similarity between an arbitrary but representative set of viruses and their human host. As a proof of concept, we used DASH to analyze the homologies between HHV-8 and human, which have been extensively documented in the past two decades. Several of the HHV-8 proteins have been reported as being of cellular origin, which DASH confirmed. Our work also confirmed functional similarities between human and both the DNA and RNA viruses, with viral genomes of various sizes and regardless of transcription strategies. Our examination of the order Mononegavirales confirmed that retroviruses have not been the only RNA viruses donating genetic material to cellular genomes. DASH also provided supporting evidence that non-retro-transcribing RNA viruses have contributed endogenized elements to the human genome.

The fractions of homologs between humans and the fifty-two viruses reported here are likely underestimates of the actual fractions. This study analyzed only the protein-coding regions of the genomes. Quite possibly, transfer of non-coding genes (*e.g.* non-coding functional RNA (rRNA, tRNA), cis-regulatory elements, etc.) may also have occurred.

In all likelihood, the proteins shared by viruses and their hosts today have been acquired through horizontal gene transfer (HGT) at some point in the past. Our functional analyses of the fifty-two human viruses suggest that genetic transfers from host to virus seem to have been predominant in the DNA viruses. Our results also show that among the viruses, the RNA viruses have been predominant donors of genetic material to the host regardless of viral transcription strategy. Our remarks of directionality are based on the annotations on the homologies, which confirm reports published elsewhere (*e.g.*[5]).

Sequence similarity methods can suggest cases of HGT, but determination of HGT directionality is more difficult to automate, because it requires a remarkably detailed phylogenetic investigation in large part directed by human input. Yet, the usefulness of a sequence-based method like DASH is in its ability to scan large amounts of data to streamline the list of protein candidates for further phylogenetics or experimental characterization. Moreover, DASH provides a platform to analyze the complete genomes of two interacting species. The analysis identifies common domains as well as those exclusive to each organism.

The current version of DASH, a prototype, allows the analysis of fifty-two reference viral genomes. The results we have shown here validate the methodology and show the potential of the pipeline to analyze other host-symbiont systems rapidly. In principle, the DASH pipeline can annotate new genomes or characterize different isolates of the same virus. DASH output can also augment the analysis of host-pathogen interaction or co-evolution data. In addition to detecting functional similarities, DASH provides sets of possibly orthologous genes for phylogenetic analysis and evolutionary gene reconstruction. DASH can also generate lists of genes and proteins potentially unique to the virus to aid rational drug design. For instance, if a peptide-binding site were unique to a virus, the design of peptide drugs would then avoid an autoimmune response in the host. Knowledge of the genetic material captured or donated by pathogens should give insights into the etiology of the diseases they cause and help inform effective drug and vaccine design.

## Competing interests

The authors declare that they have no competing interests.

## Authors’ contributions

Both authors drafted the manuscript. MWG created the DASH pipeline and performed the analysis. JLS designed the web interface and supervised the study. Both authors read and approved the final manuscript.

## Supplementary Material

Additional file 1: Table S1 Human and HHV-8 Homologs identified by PSI-BLAST and HMMER. Click here for file
