# Supplementary material for: Domain analysis of symbionts and hosts (DASH) in a genome-wide survey of pathogenic human viruses
Source: BMC Res Notes. 2013 May 24;6:209. doi: 10.1186/1756-0500-6-209 (PMC3672079; doi:10.1186/1756-0500-6-209)
Supplement: Additional file 1: Table S1 — Human and HHV-8 Homologs identified by PSI-BLAST and HMMER. [file 1756-0500-6-209-S1.docx]

**Additional File 1 – Human and HHV-8 Homologs identified by PSI-BLAST and HMMER.**

| **HHV-8 Gene** | **HHV-8 protein gi** | **Function** | **HMMER** | | | **PSI-BLAST** | | |
| --- | --- | --- | --- | --- | --- | --- | --- | --- |
|  |  |  | **HHV-8 E-value** | **Human E-value** | **Human E-value** | | **Human sequence gi** |  |
| ORF71/K13 | 139472802 | CASP8 and FADD-like apoptosis regulator | 3e-12 | 2e-18 | 5e-48 | | 62088780 |  |
| ORF74 | 139472805 | 7 transmembrane receptor | 4e-14 | 6e-94 | 9e-161 | | 183979982 |  |
| ORF9 | 139472809 | DNA polymerase | 2e-141 | 5e-134 | 0 | | 181622 |  |
| K2 | 139472811 | Interleukin-6/G-CSF/MGF family | 8e-11 | 2e-57 | 3e-62 | | 10834984 |  |
| ORF70 | 139472812 | Thymidylate synthase | 3e-109 | 3e-113 | 0e+00 | | 4507751 |  |
| K4.1 | 139472813 | Small cytokine, interleukin-8 like | 3e-06 | 3e-15 | 3e-05 | | 12641915 |  |
| K4 | 139472814 | Small cytokine, interleukin-8 like | 1e-13 | 3e-15 | 3e-12 | | 10834978 |  |
| K5 | 139472815 | RING-variant domain | 6e-07 | 1e-06 | 8e-52 | | 19263985 |  |
| ORF16 | 139472817 | Apoptosis regulator, Bcl-2 family | 4e-05 | 4e-24 | 3e-36 | | 72198189 |  |
| ORF46 | 139472838 | Uracil DNA glycosylase | 7e-15 | 9e-20 | 3e-136 | | 6224979 |  |
| ORF54 | 139472845 | dUTPase | 1e-07 | 8e-40 | 3e-25 | | 2443580 |  |
| ORF56 | 139472847 | Herpesviridae UL52/UL70 DNA primase | 1e-12 | 1e-07 | Not found | | Not found |  |
| vIRF-4/K10/K10.1 | 139472849 | Interferon regulatory factor | 5e+00 | 2e-59 | Not found | | Not found |  |
| vIRF-3/K10.5/K10.6 | 139472850 | Interferon regulatory factor | 1e-03 | 2e-59 | Not found | | Not found |  |
| vIRF-2/K11/K11.1 | 139472851 | Interferon regulatory factor | 6e-01 | 2e-59 | Not found | | Not found |  |
| ORF60 | 139472853 | Ribonucleotide reductase, small chain | 2e-87 | 2e-116 | 0 | | 260064013 |  |
| KCP/ORF4 | 139472860 | Complement control protein | 1e-02 | 1e-04 | 2e-122 | | 62898986 |  |
| ORF2 | 139472863 | Dihydrofolate reductase | 5e-22 | 6e-25 | 6e-98 | | 4503323 |  |
| K3 | 139472864 | RING-variant domain | 3e-06 | 1e-06 | 5e-59 | | 19263985 |  |
| K6 | 139472866 | Small cytokine, interleukin-8 like | 2e-13 | 3e-15 | 8e-13 | | 10834978 |  |
| K9 | 139472878 | Interferon regulatory factor | 2e-16 | 2e-59 | 3e-110 | | 334883190 |  |
| ORF61 | 139472880 | Ribonucleotide reductase, large chain | 6e-124 | 7e-211 | 0 | | 5006420 |  |
| ORF72 | 139472885 | Cyclin | 3e-21 | 4e-39 | 4e-122 | | 4757930 |  |
| ORF75 | 139472887 | CobB/CobQ-like glutamine amidotransferase, AIR synthase | 2e-42 | 1e-87 | Not found | | Not found |  |
